# Socioecology and Prevalence of SARS‐CoV‐2 Infection in *Quilombolas* Living in the Brazilian Amazon

**DOI:** 10.1002/ajhb.70055

**Published:** 2025-05-03

**Authors:** Keise Adrielle Santos Pereira, Lilian Natalia Ferreira de Lima, Bruno José Sarmento Botelho, Carlos Neandro Cordeiro Lima, Wiliane Freire Pinheiro, Victor Martins Eleres, Wandrey Roberto dos Santos Brito, Bernardo Cintra dos Santos, Aline Cecy Rocha de Lima, Felipe Teixeira Lopes, Isabella Nogueira Abreu, Maria Karoliny da Silva Torres, Sandra Souza Lima, Jacqueline Cortinhas Monteiro, Andrea Nazaré Monteiro Rangel da Silva, João Farias Guerreiro, Izaura Maria Vieira Cayres Vallinoto, Hilton P. Silva, Antonio Carlos Rosário Vallinoto, Rosimar Neris Martins Feitosa

**Affiliations:** ^1^ Laboratório de Virologia Universidade Federal Do Pará Belém Pará Brazil; ^2^ Instituto de Ciências Biológicas, Programa de Pós‐graduação Em Biologia de Agentes Infecciosos e Parasitários Universidade Federal Do Pará Belém Pará Brazil; ^3^ Universidade Do Estado Do Tocantins Augustinópolis Tocantins Brazil; ^4^ Laboratório de Genética Humana e Médica Universidade Federal Do Pará Belém Pará Brazil; ^5^ Programa de Pós‐Graduação Em Antropologia e Programa de Pós‐Graduação Em Saúde Coletiva Na Amazônia Universidade Federal Do Pará Belém Brazil; ^6^ Centro de Estudos Avançados Multidisciplinares Universidade de Brasília Brasília Brazil; ^7^ Research Center for Anthropology and Health University of Coimbra Coimbra Portugal

**Keywords:** Amazon, COVID‐19, health of the Black population, public policy, socioecology, Amazônia, Políticas Públicas, Saúde da população negra, Socioecologia, Amazonía, Políticas Públicas, Socioecología, Salud de la población negra

## Abstract

**Objectives:**

This cross‐sectional study presents socioecological, epidemiological aspects, and the seroprevalence of immunoglobulin G (IgG) against severe acute respiratory syndrome coronavirus 2 (SARS‐CoV‐2) in a group of *quilombola* (afro‐derived) communities in the states of Pará and Tocantins, in the Brazilian Amazon, to evaluate the impact of SARS‐CoV‐2 prevalence among them.

**Methods:**

A total of 551 individuals participated. The detection of anti‐SARS‐CoV‐2 antibodies was performed using an enzyme immunoassay. Socioeconomic and ecological data was collected from all participants 7 years of age or older who were not previously vaccinated.

**Results:**

The seroprevalence of antibodies in both states was 40.7% and was associated with factors such as age group, contact with infected individuals, and being in lockdown inside the *quilombos*. In Pará, a statistically significant association was observed between seroprevalence and females, and the age group of 12–18 years. In addition, seroprevalence in Pará was higher than in Tocantins, and the reported use of masks was a protective factor, while in Tocantins, the reported use of masks was associated with the presence of antibodies. There was no association between the prevalence of antibodies and the presence of COVID‐19 symptoms in Pará. However, in Tocantins, diarrhea and loss of taste were associated with infection.

**Conclusions:**

*Quilombola* are highly vulnerable groups due to the long history of enslavement in Brazil. This is the first investigation of SARS‐CoV‐2 seroprevalence and its impact in these groups in the Amazon. The study helps us to understand the relationship of socioecological differences, behavioral characteristics, and the dynamics of viral transmission associated with the risk of infection by SARS‐CoV‐2 among traditional populations, and can be useful to the planning of more culturally adequate public health policies for future epidemics.

## RESUMO

**Objetivos:**

Este estudo transversal apresenta aspectos socioecológicos, epidemiológicos e a soroprevalência de imunoglobulinas G (IgG) contra o Coronavírus da Síndrome Respiratória Aguda Grave 2 (SARS‐CoV‐2) em um grupo de comunidades *quilombolas* nos estados do Pará e Tocantins, na Amazônia Brasileira, para avaliar o impacto do SARS‐CoV‐2 nesses grupos.

**Métodos:**

Um total de 551 indivíduos participou do estudo. A detecção de anticorpos anti‐SARS‐CoV‐2 foi realizada utilizando um ensaio imunoenzimático. Dados socioeconômicos e ecológicos foram coletados de todos os participantes de 7 anos ou mais que não haviam sido previamente vacinados.

**Resultados:**

A soroprevalência de anticorpos em ambos os estados foi de 40,7% e essa prevalência foi associada a fatores como a faixa etária, contato com indivíduos infectados e ficar em *lockdown* nos *quilombos*. No Pará, foi observada associação estatisticamente significante da soroprevalência com o sexo feminino e a faixa etária de 12 a 18 anos. Além disso, a soroprevalência no Pará foi maior que no Tocantins e o uso relatado de máscara foi um fator de proteção, enquanto em Tocantins, o uso relatado de máscara foi associado à presença de anticorpos. Não houve associação entre a prevalência de anticorpos e a presença de sintomas de COVID‐19 no Pará. Porém, em Tocantins, a diarreia e a perda de paladar foram considerados fatores de risco para a infecção.

**Conclusões:**

*Quilombolas* são grupos altamente vulnerabilizados devido à longa história de escravidão no Brasil. Esta é a primeira investigação de seroprevalência para SARS‐CoV‐2 e seu impacto nesses grupos na Amazônia. O estudo ajuda a compreender os impactos das diferenças ecológicas, características comportamentais e dinâmicas de transmissão viral associadas ao risco de infecção por SARS‐CoV‐2 entre grupos tradicionais, e será útil no planejamento de políticas públicas de saúde mais culturalmente adequadas para epidemias futuras.

## RESUMEN

**Objetivos:**

Este estudio transversal presenta aspectos socioecológicos y epidemiológicos y la seroprevalencia de inmunoglobulinas G (IgG) frente al Coronavirus 2 del Síndrome Respiratorio Agudo Severo (SARS‐CoV‐2) en un grupo de comunidades *quilombolas* de los estados de Pará y Tocantins, en la Amazonía brasileña, para evaluar el impacto del SARS‐CoV‐2 en estos grupos.

**Métodos:**

Participaron en el estudio 551 individuos. La detección de anticuerpos anti‐SARS‐CoV‐2 se realizó mediante un ensayo de inmunoabsorción ligado a enzimas. Se recogieron datos socioeconómicos y ecológicos de todos los participantes de 7 años o más que no habían sido vacunados previamente.

**Resultados:**

La seroprevalencia de anticuerpos en ambos estados fue de 40,7% y esta prevalencia se asoció a factores como el grupo de edad (12 a 18 años), el contacto con individuos infectados y el encierro en el territorio de *quilombos*. En Pará, se observó una asociación estadísticamente significativa entre la seroprevalencia y el sexo femenino y el grupo de edad de 12 a 18 años. Además, la seroprevalencia en Pará fue mayor que en Tocantins y el uso reportado de mascarillas fue un factor protector, mientras que en Tocantins, el uso reportado de mascarillas se asoció con la presencia de anticuerpos. No hubo asociación entre la prevalencia de anticuerpos y la presencia de síntomas de COVID‐19 en Pará. Sin embargo, en Tocantins, la diarrea y la pérdida del gusto se consideraron factores de riesgo para la infección.

**Conclusiones:**

Los *quilombolas* son grupos altamente vulnerables debido a la larga historia de esclavitud en Brasil. Esta es la primera investigación sobre la seroprevalencia del SARS‐CoV‐2 y su impacto en estos grupos en la Amazonía. El estudio ayuda a comprender los impactos de las diferencias ecológicas, las características de comportamiento y la dinámica de transmisión viral asociada con el riesgo de infección por SARS‐CoV‐2 entre los grupos tradicionales, y será útil para planificar políticas de salud pública más culturalmente apropiadas para futuras epidemias.

## Introduction

Between the 16th and the 19th centuries, Brazil engaged in the large‐scale enslavement and trade of enslaved people from the African Continent (Graham 2002). Upon arrival, many resisted the forced labor in various ways. The term *Quilombo* was first used to describe both a physical community and a resistance movement against the slavery system in Brazil which lasted from circa 1540 to 1888, when it was legally abolished. These communities, of Black ‘runaways’ from farms and plantations, formed parallel centers of power, production, and social organization that continued to oppose oppressive and racist practices against the Brazilian Black population, even long after abolition of slavery (O'Dwyer 2002; Salles 2005).

Although historically there have been many definitions of *Quilombos* and *Quilombolas* (Amaral 2009; Arruti 2008; Paiva et al. 2023; Salles 2005), currently, *quilombolas* are those who live in the *Quilombo* territory or are their descendants. These communities are located in all regions of Brazil, especially but not exclusively rural areas, and many still maintain a degree of isolation due to their geographic location (Gontijo et al. 2014). Most of the communities originated one or two centuries ago. They are often located in remote areas such as forests and hinterlands. They consist of small or large interdependent groups living in spatially contiguous dwellings, with strong family and community ties, and have adapted to the natural environments of Brazil (IBGE 2020; O'Dwyer 2002). In general, these self‐identified *Quilombos* are characterized by low socioeconomic status, high frequency of chronic and infectious diseases, racial discrimination, and limited access to sanitation and health services. They are among the most vulnerable traditional groups in the country (Cavalcante and Silva 2019; Paiva et al. 2023; Santos et al. 2020; Silva et al. 2020). According to the 2022 Brazilian national census, there are over 8441 *quilombola* localities, encompassing more than 1.3 million people (MIR 2022), making this the largest ethnic minority group in the country. Most of their territories are not officially recognized by the government, making them vulnerable to eviction and violence by large farmers and land invaders. These conflicts directly impact environmental preservation, as these traditional populations, along with indigenous groups, contribute to protecting the Amazon rainforests and other natural ecosystems. Structural racism, land conflict, social discrimination, and violence in the Amazon region have made these populations more vulnerable to the COVID‐19 pandemic (Arruti et al. 2021; Carvalho et al. 2021; Silva and Souza 2021).

On February of 2021, *quilombola* organizations, led by the National Coordination for the Articulation of Rural Black Quilombo Communities (*Coordenação Nacional de Articulação das Comunidades Negras Rurais Quilombolas*—CONAQ), won a Federal Class Action in the Supreme Court (ADPF 742). This action mandated the federal government to fulfill its constitutional duty to protect the more vulnerable citizens. However, the government at the time did not support the causes of traditional forest peoples, and racism and discrimination had already resulted in thousands of infections and hundreds of deaths (Arruti et al. 2021; Brasil 2022; Carvalho et al. 2021).

In response to the pandemic and the mandate of ADPF 742 to assist in the fight against the coronavirus disease 2019 (COVID‐19), the Brazilian Institute of Geography and Statistics (IBGE) expedited the release of the Territorial Base of the 2020 Demographic Census and made available the *quilombo* locations identified up to 2019 (IBGE 2020). According to that IBGE data, there were approximately 890 communities in the North region, of which 516 are distributed across 65 municipalities in Pará, and 84 communities in 31 municipalities in Tocantins (IBGE 2020).

According to the COVID‐19 Observatory of Quilombos, created by CONAQ in partnership with the Socioenvironmental Institute (*Instituto Socioambiental*—ISA), approximately 5666 *quilombola* cases and 301 deaths were registered until January 2022, the last information available. Due to the very limited testing in *quilombola* communities, these numbers likely represent only a fraction of the actual number of COVID‐19 cases and deaths among them. CONAQ stated that the invisibility of the disease in *quilombos* was a result of persistent structural racism in Brazil. This racism led to high underreporting of data on disease transmission and deaths, especially among the most isolated and elderly, who faced greater difficulty accessing health services (CONAQ 2020).

Since the first cases of COVID‐19 were reported in Wuhan city, Hubei Province, China, in December 2019, the severe acute respiratory syndrome coronavirus 2 (SARS‐CoV‐2) spread rapidly worldwide, causing a pandemic (WHO 2020; Zhu et al. 2020). Globally, approximately 753 823 259 cases and 6 814 976 deaths from COVID‐19 had been confirmed by February 2023 (WHO 2023). The disease disproportionately affects the most socially and economically vulnerable groups, such as traditional communities in developing countries and minority groups in developed countries (Palamim et al. 2020; Polidoro et al. 2021; Tai et al. 2021).

In Brazil, as of February 2023, 36 837 943 cases and 697 200 deaths from COVID‐19 had been confirmed (Brasil 2023), making it the second country with the highest pandemic mortality rate, behind only the United States. In the state of Pará, in the Amazon basin, the number of cases reached 866 600 with 19 004 deaths. In the state of Tocantins, also an Amazonian state, 364 313 cases with 4228 deaths were reported (SESPA 2023; SES‐TO 2023). The space–time distribution of cases and deaths from the disease since 2020 has followed a heterogeneous pattern, highlighting the sociodemographic and health infrastructure inequalities existing in the country (Brasil 2022). Therefore, in close association with increased socioeconomic inequities, COVID‐19 spread unevenly across all regions, primarily affecting the most vulnerable groups (ABRASCO 2021; Varga et al. 2020).

Considering this scenario, the absence of seroepidemiological data for *quilombola* populations and the underreporting of cases have obscured the true situation experienced by these communities. According to Vallinoto et al. (2020), seroepidemiological studies are extremely relevant, as they allow the analysis of seroprevalence and antibody titers, as well as the evaluation of epidemiological aspects of exposure risk in vulnerable populations. The present study aimed to describe the impact of the socioecological conditions, the seroprevalence of anti‐SARS‐CoV‐2 IgG antibodies, and the epidemiological aspects of exposure to the virus in Amazonian *quilombo* communities living in the states of Pará and Tocantins, and to analyze the risk factors associated with COVID‐19 among these rural communities.

## 1 Materials and Methods

### 1.1 Study Design and Sampling

**FIGURE 1:**
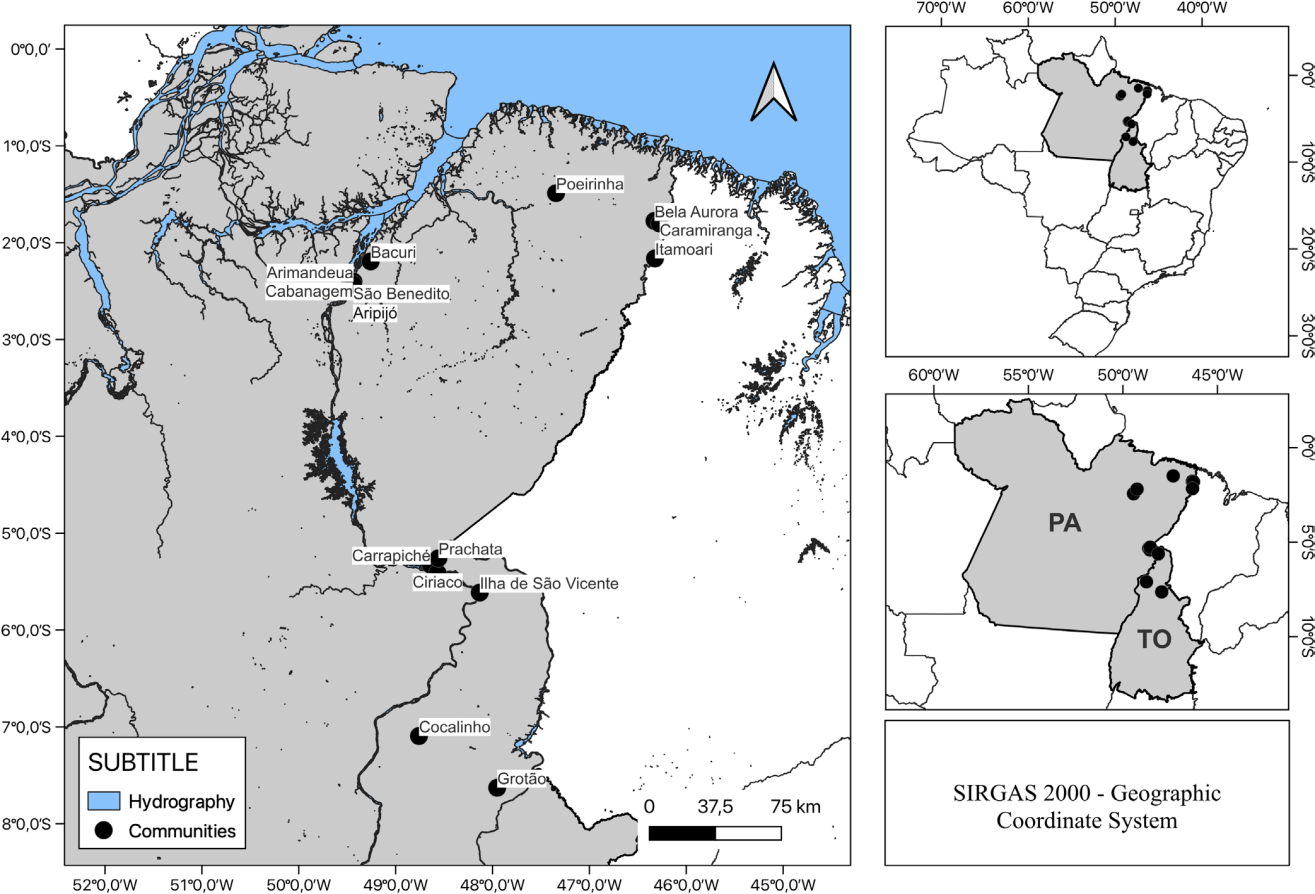
Geographic location of *quilombola* communities. Communities in the state of Pará: Poeirinha, municipality of Bonito; Bela Aurora, Camiranga, and Itamoari, municipality of Cachoeira do Piriá; Arimandeua, Aripijó, Bacuri, Cabanagem, and São Benedito, municipality of Cametá. Communities in the state of Tocantins: Carrapiché, Ciriaco, and Prachata, municipality of Esperantina; São Vicente Island, municipality of Araguatins; Cocalinho, municipality of Santa Fé do Araguaia; Groton, Philadelphia County. PA, Pará; TO, Tocantins.

This cross‐sectional seroepidemiology surveillance study was conducted in *quilombola* communities located in the states of Pará and Tocantins. The research team visited nine (9) communities in Pará between September 2020 and April 2021 (at the end of the first wave of COVID‐19 and at the peak of the second wave in Brazil): Poeirinha (*n* = 20), Bela Aurora (*n* = 34), Camiranga (*n* = 72), Itamoari (*n* = 73), Arimandeua (*n* = 37), Aripijó (*n* = 28), Bacuri (*n* = 10), Cabanagem (*n* = 15), São Benedito (*n* = 63); and six (6) communities were visited in Tocantins between February and July 2021 (beginning and end of the second wave of COVID‐19): Carrapiché (*n* = 29), Ciriáco (*n* = 20), Prachata (*n* = 25), São Vicente Island (*n* = 79), Cocalinho (*n* = 24), and Grotão (*n* = 22) (Figure 1). A total of 551 individuals, aged between 7 and 91 years, participated in the study.

### 1.2 Ethical Aspects

The project was submitted and approved by the Human Research Ethics Committee of the Health Sciences Institute of the Federal University of Pará (process number 4.167.592) and by the Human Research Ethics Committee of the State University of Tocantins (process number 5.140.703). This was done in compliance with all ethical and scientific requirements, respecting Resolution No. 466/12 of the Brazilian Ministry of Health.

The consent of the communities to conduct the study was obtained through their leaders. All study participants were voluntary and signed an informed consent form (TCLE). Participants under 18 years of age signed an agreement consent form (TALE) accompanied by the TCLE signed by their legal guardians. Individuals vaccinated against SARS‐CoV‐2 (one or two doses), those who did not sign the consent form, those who did not respond to the epidemiological questionnaire, and those under 7 years of age were excluded from the study.

### 1.3 Data Collection

Data collection was performed by a multidisciplinary health team. In Pará, the field work was conducted in partnership with the Secretary of Health of the State of Pará and the Laboratory of Human and Medical Genetics of the Federal University of Pará. In Tocantins, data collection was carried out in partnership with the State University of Tocantins team. All safeguard procedures for the protection of the research team and the participants in relation to infection by the SARS‐CoV‐2 virus were followed, adhering to World Health Organization (WHO) guidelines. After signing the TCLE, individuals were interviewed using a structured questionnaire. The questionnaire included questions about sociodemographic, ecological, behavioral, and clinical characteristics.

The following data were collected from each participant: sociodemographic data, including (1) sex, age, family income, education, self‐reported race/skin color following the Brazilian Census Bureau (IBGE) procedures; (2) behavioral information, including preventive measures such as wearing masks, hand hygiene, social distancing, contact with people infected with SARS‐CoV‐2, and travel outside the community; and (3) information related to COVID‐19 infection, such as the presence of symptoms (fever, headache, runny nose, cough, sore throat, body ache, abdominal pain, diarrhea, nausea, vomit, loss of smell, loss of taste, shortness of breath, and fatigue). All data were stored on the UFPA Virology Laboratory's local server using EPI Info version 7.2.4 software.

### 1.4 Collection, Processing, and Storage of Biological Samples

After obtaining the informed consent form and the completed questionnaire, peripheral venous blood samples (10 mL) were collected by venipuncture in two vacuum tubes containing EDTA. Subsequently, the samples were centrifuged, and plasma and leukocytes were stored at −70°C.

### 1.5 Serological Analysis

An ELISA‐type immunoenzymatic assay (Euroimmun, Luebeck, Germany) was used for the detection of anti‐SARS‐CoV‐2 IgG antibodies in plasma. Procedures were performed according to the manufacturer's protocol. Samples were classified as reactive (ratio ≥ 1.1), nonreactive (ratio < 0.8), or indeterminate (ratio ≥ 0.8 and < 1.1), according to the manufacturer. The assay used has a clinical sensitivity of 75%–93.8% (> 10 to 20 days to ≥ 21 days after disease onset) and a specificity of 99.6% for IgG antibodies, according to the manufacturer's guidelines.

### 1.6 Statistical Analysis

Socioecological and epidemiological characteristics were described using simple frequency and percentages. To identify associations between the studied characteristics and infection, the chi‐square test and the *G* test were used. Variables with a *p* value less than or equal to 0.25 in the bivariate analysis were included in the Multiple Logistic Regression analysis. In all analyses, the adopted statistical significance level was set at 0.05. The analyses were performed using the BioEstat 5.3 program, and the graph was generated in GraphPad Prism version 8.0.

## 2 Results

This study included 551 individuals from 15 *quilombola* communities in the states of Pará (*n* = 352) and Tocantins (*n* = 199). The sample comprised individuals of both sexes (340 women and 211 men) with ages ranging from 7 to 91 years (median 31 years, IIIQ 48 years, SD = 19.2). None of the individuals included in the study had been previously vaccinated against COVID‐19 at the time of data collection. Serological results indicated that 210 (38.1%) individuals were reactive for anti‐SARS‐CoV‐2 IgG antibodies, 306 (55.5%) were nonreactive, and 35 (6.4%) had indeterminate results.

**FIGURE 2:**
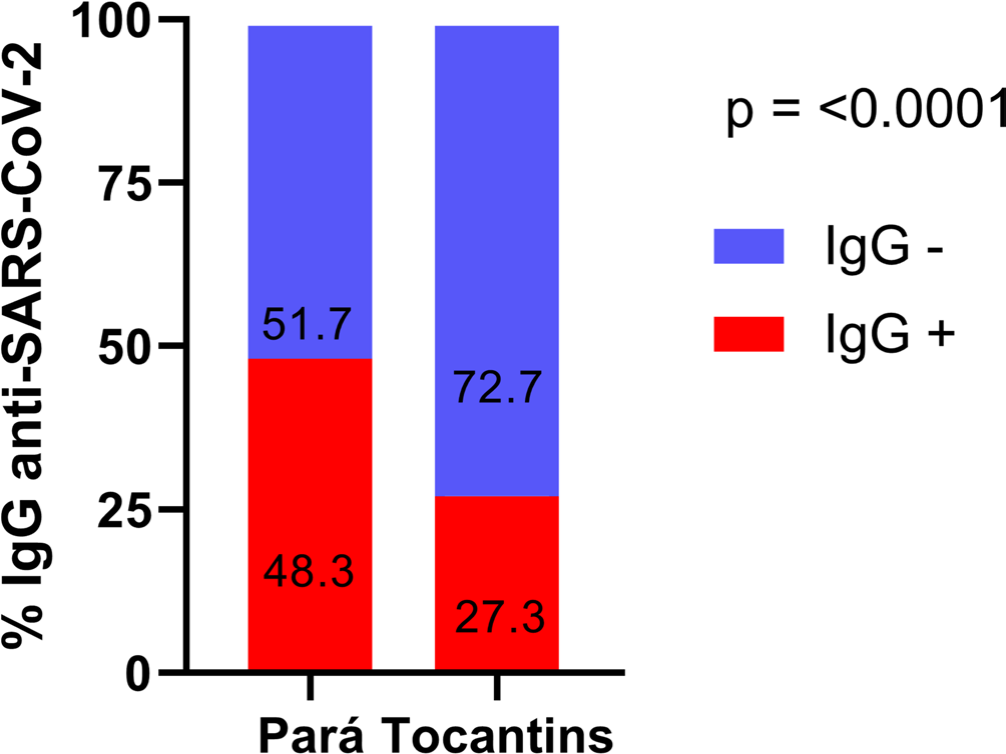
Seroprevalence of IgG‐anti‐SARS‐CoV‐2 antibodies in *quilombola* communities in the states of Pará and Tocantins.

A total of 516 (93.6%) individuals were included in the final statistical analysis, excluding those with indeterminate results. In this general analysis, the seroprevalence of anti‐SARS‐CoV‐2 IgG antibodies observed in both states was 40.7%. In the *quilombos* of Pará, the seroprevalence was 48.3% (95% CI: 42.9–53.7), while in Tocantins, the prevalence was 27.3% (95% CI: 20.9–33.7), showing a statistically significant difference (*p* ≤ 0.0001) (Figure 2).

In the bivariate analysis, no statistically significant association was observed between any of the sociodemographic characteristics and seroprevalence in the *quilombos* of the two regions. However, in Pará, individuals aged 12–18 years (23.3%; *p* = 0.0162) had a higher risk of infection, while no significant association was found for the other variables. In Tocantins, there was no significant association for any of the sociodemographic characteristics (Table 1). Additionally, the analysis showed high prevalence of antibodies anti‐SARS‐CoV‐2 in females as well as in individuals in the age group of 30–59 years, but without statistical significance.

**TABLE 1:** Sociodemographic characteristics associated with the seroprevalence of SARS‐CoV‐2 in quilombola communities in the states of Pará and Tocantins.

Variables, *N* (%)	Pará and Tocantins			Pará			Tocantins		
	Reactive, *n* = 210 (40.7%)	Nonreactive, *n* = 306 (59.3%)	*p*	Reactive, *n* = 159 (48.3%)	Nonreactive, *n* = 170 (51.7%)	*p*	Reactive, *n* = 51 (27.3%)	Nonreactive, *n* = 136 (72.7%)	*p*
Sex			0.1465^a^			0.0722^a^			0.9879^a^
Female	137 (65.2)	179 (58.5)		107 (67.3)	97 (57.1)		30 (58.8)	82 (60.3)	
Male	73 (34.8)	127 (41.5)		52 (32.7)	73 (42.9)		21 (41.2)	54 (39.7)	
Age group			0.3363^a^			0.0162^a^			
7–11	15 (7.1)	29 (9.5)		12 (7.5)	14 (8.2)		3 (5.9)	15 (11.0)	0.4811^b^
12–18	48 (22.9)	49 (16.0)		37 (23.3)	18 (10.6)		11 (21.6)	31 (22.8)	
19–29	39 (18.6)	61 (19.9)		25 (15.7)	31 (18.2)		14 (27.5)	30 (22.1)	
30–59	81 (38.6)	130 (42.5)		62 (39.0)	89 (52.4)		19 (37.3)	41 (30.1)	
60 years or older	25 (11.9)	34 (11.1)		22 (13.8)	18 (10.6)		3 (5.9)	16 (11.8)	
No information^c^	2 (1.0)	2 (1.0)		1 (0.6)	0 (0.0)		1 (2.0)	3 (2.2)	
Family income			0.8206^a^			0.1454^a^			0.5747^b^
< 1	114 (54.3)	189 (61.8)		89 (56.0)	110 (64.7)		25 (49.0)	79 (58.1)	
1	64 (30.5)	82 (26.8)		45 (28.3)	38 (22.4)		19 (37.3)	44 (32.4)	
2 or more	18 (8.6)	19 (6.2)		12 (7.5)	7 (4.1)		6 (11.8)	12 (8.8)	
No information^c^	14 (6.7)	16 (5.2)		13 (8.2)	15 (8.8)		1 (2.0)	1 (0.7)	
Education			0.0818^a^			0.1681^b^			0.4217^b^
Illiterate	6 (2.9)	8 (2.6)		4 (2.5)	1 (0.6)		2 (3.9)	7 (5.1)	
Literate	82 (39.0)	145 (47.4)		64 (40.3)	80 (47.1)		18 (35.3)	65 (47.8)	
Primary education	13 (6.2)	28 (9.2)		8 (5.0)	15 (8.8)		5 (9.8)	13 (9.6)	
High school	82 (39.0)	85 (27.8)		62 (39.0)	51 (30.0)		20 (39.2)	34 (25.0)	
University education	17 (8.1)	26 (8.5)		13 (8.2)	13 (7.6)		4 (7.8)	13 (9.6)	
No information^c^	10 (4.8)	14 (4.6)		8 (5.0)	10 (5.9)		2 (3.9)	4 (2.9)	
Skin color			0.5052^b^			0.2902^b^			
Yellow	1 (0.5)	5 (1.6)		0 (0.0)	2 (1.2)		1 (2.0)	3 (2.2)	0.7427^b^
White	14 (6.7)	17 (5.6)		13 (8.2)	11 (6.5)		1 (2.0)	6 (4.4)	
Black	118 (56.2)	164 (53.6)		88 (55.3)	77 (45.3)		30 (58.8)	87 (64.0)	
Brown	73 (34.8)	117 (38.2)		55 (34.6)	78 (45.9)		18 (35.3)	39 (28.7)	
No information^c^	4 (1.9)	3 (1.0)		3 (1.9)	2 (1.2)		1 (2.0)	1 (0.7)	

^a^ Chi‐square test.

^b^ Test *G*.

^c^ Not included in statistics.

Regarding behavioral characteristics, reported contact with known infected individuals was significantly associated with SARS‐CoV‐2 infection (35.2%; *p* = 0.0451). Furthermore, the seroprevalence of antibodies was higher among individuals who stayed in lockdown within the *quilombo* area (85.7%; *p* = 0.0243) compared to those who were not in lockdown (13.3%) when we analyzed all the quilombos together. However, this significance was no longer observed when we analyzed the quilombos by state. Commuting, travel, mask use, and hand washing were not significantly associated with infection in the overall sample. In the *quilombos* of Pará, most individuals who reported using a mask when leaving their homes tested negative for anti‐SARS‐CoV‐2 IgG antibodies (75.9%; *p* = 0.0165), with no significant association with the other variables. In the communities of Tocantins, mask use (82.4%; *p* = 0.0166) and hand washing (80.4%; *p* = 0.0218) were associated with the presence of anti‐SARS‐CoV‐2 IgG antibodies, without a significant association with the other variables (Table 2).

**TABLE 2:** Behavioral characteristics associated with the seroprevalence of anti‐SARS‐CoV‐2 IgG antibodies in quilombola communities in the states of Pará and Tocantins.

Variables, *N* (%)	Pará and Tocantins			Pará			Tocantins		
	Reactive, *n* = 210 (40.7%)	Nonreactive, *n* = 306 (59.3%)	*p*	Reactive, *n* = 159 (48.3%)	Nonreactive, *n* = 170 (51.7%)	*p*	Reactive, *n* = 51 (27.3%)	Nonreactive, *n* = 136 (72.7%)	*p*
Contact with infected person			0.0451^a^			0.1257^a^			0.8465^a^
Yes	74 (35.2)	82 (26.8)		61 (38.4)	51 (30.0)		13 (25.5)	31 (22.8)	
No	134 (63.8)	223 (72.9)		96 (60.4)	118 (69.4)		38 (74.5)	105 (77.2)	
No information^b^	2 (1.0)	1 (0.3)		2 (1.2)	1 (0.6)		0 (0.0)	0 (0.)	
Stayed in lockdown in the *quilombo*			0.0243^a^			0.0781^a^			0.2856^a^
Yes	180 (85.7)	238 (77.8)		136 (85.5)	132 (77.6)		44 (86.3)	106 (77.9)	
No	28 (13.3)	66 (21.6)		21 (13.2)	36 (21.2)		7 (13.7)	30 (22.1)	
No information^b^	2 (1.0)	2 (0.7)		2 (1.3)	2 (1.2)		0 (0.0)	0 (0.)	
Left the *quilombo* during lockdown			0.4767^a^			0.2782^a^			0.7422^a^
Yes	164 (78.1)	247 (80.7)		121 (76.1)	137 (80.6)		43 (84.3)	110 (80.9)	
No	44 (21.0)	55 (18.0)		36 (22.6)	29 (17.1)		8 (15.7)	26 (19.1)	
No information^b^	2 (1.0)	4 (1.3)		2 (1.3)	4 (2.3)		0 (0.0)	0 (0.)	
Traveled			0.9231^a^			0.9589^a^			0.7707^a^
Yes	75 (35.7)	108 (35.3)		54 (34.0)	57 (33.5)		21 (41.2)	51 (37.5)	
No	132 (62.9)	197 (64.4)		102 (64.2)	112 (65.9)		30 (58.8)	85 (62.5)	
No information^b^	3 (1.4)	1 (0.3)		3 (1.9)	1 (0.6)		0 (0.0)	0 (0.)	
Use of mask when going out			0.9399^a^			0.0165^a^			0.0166^a^
Always	144 (68.6)	209 (68.3)		102 (64.2)	129 (75.9)		42 (82.4)	80 (58.8)	
Rarely	52 (24.8)	75 (24.5)		43 (27.0)	27 (15.9)		9 (17.6)	48 (35.3)	
No information^b^	14 (6.6)	22 (7.2)		14 (8.8)	14 (8.2)		0 (0.0)	8 (5.9)	
Handwashing			0.5036^a^			0.9411^a^			0.0218^c^
Always	153 (72.9)	212 (69.3)		112 (70.4)	117 (68.8)		41 (80.4)	95 (69.9)	
Only sometimes	38 (18.1)	65 (21.2)		31 (19.5)	33 (19.4)		7 (13.7)	32 (23.5)	
Rarely	8 (3.8)	16 (5.2)		8 (5.0)	7 (4.1)		0 (0.0)	9 (6.6)	
No information^b^	11 (5.2)	13 (4.2)		8 (5.0)	13 (7.6)		3 (5.9)	0 (0.0)	

^a^ Chi‐square test.

^b^ Not included in statistics.

^c^ Test *G*.

In Pará, no symptoms were associated with the seroprevalence of anti‐SARS‐CoV‐2 antibodies. However, in Tocantins, symptoms such as fever (19.6%; *p* = 0.0148), headache (25.5%; *p* = 0.0198), body pain (25.5%; *p* = 0.0198), diarrhea (15.7%; *p* = 0.0128), loss of smell, and loss of taste (11.8; *p* = 0.0060) were more prevalent among the reactive individuals (Table 3).

**TABLE 3:** Frequency of COVID‐19 symptoms according to the seroprevalence of anti‐SARS‐CoV‐2 IgG in quilombo communities in the states of Pará and Tocantins.

Symptoms	Pará and Tocantins			Pará			Tocantins		
	Reactive *n* = 210 (40.7%)	Nonreactive *n* = 306 (59.3%)	*p*	Reactive *n* = 159 (48.3%)	Nonreactive *n* = 170 (51.7%)	*p*	Reactive *n* = 51 (27.3%)	Nonreactive *n* = 136 (72.7%)	*p*
Fever	29 (13.8)	32 (105)	0.3078^a^	19 (11.9)	22 (12.9)	0.9163^a^	10 (19.6)	10 (7.4)	0.0148^b^
Headache	42 (20.0)	64 (20.9)	0.8872^a^	29 (18.2)	46 (27.1)	0.0760^a^	13 (25.5)	18 (13.2)	0.0198^a^
Runny nose	42 (20.0)	57 (18.6)	0.7832^a^	33 (20.8)	38 (22.4)	0.8274^a^	9 (17.6)	19 (14.0)	0.3847^a^
Cough	37 (17.6)	45 (14.7)	0.4433^a^	28 (17.6)	27 (15.9)	0.7857^a^	9 (17.6)	18 (13.2)	0.3183^a^
Sore throat	28 (13.3)	39 (12.7)	0.9209^a^	19 (11.9)	28 (16.5)	0.3109^a^	9 (17.6)	11 (8.1)	0.0521^b^
Body ache	37 (17.6)	52 (17.0)	0.9472^a^	24 (15.1)	34 (20.0)	0.3067^a^	13 (25.5)	18 (13.2)	0.0198^a^
Abdominal pain	20 (9.5)	20 (6.5)	0.2804^a^	14 (8.8)	13 (7.6)	0.8560^a^	6 (11.8)	7 (5.1)	0.1289^b^
Diarrhea	18 (8.6)	20 (6.5)	0.4851^a^	10 (6.3)	14 (8.2)	0.6411^a^	8 (15.7)	6 (4.4)	0.0128^b^
Nausea	16 (7.6)	22 (7.2)	0.9905^a^	10 (6.3)	13 (7.6)	0.7900^a^	6 (11.8)	9 (6.6)	0.2476^b^
Vomit	11 (5.2)	8 (2.6)	0.1879^a^	6 (3.8)	5 (2.9)	0.9101^b^	5 (9.8)	3 (2.2)	0.0447^b^
Loss of smell	28 (13.3)	21 (6.9)	0.0209^a^	22 (13.8)	19 (11.2)	0.5735^a^	6 (11.8)	2 (1.5)	0.0060^b^
Loss of taste	24 (11.4)	19 (6.2)	0.0517^a^	18 (11.3)	17 (10.0)	0.8342^a^	6 (11.8)	2 (1.5)	0.0060^b^
Shortness of breath	15 (7.1)	20 (6.5)	0.9274^a^	12 (7.5)	14 (8.2)	0.9787^a^	3 (5.9)	6 (4.4)	0.7801^b^
Fatigue	30 (14.3)	27 (8.8)	0.0716^a^	24 (15.1)	20 (11.8)	0.4687^a^	6 (11.8)	7 (5.1)	0.1289^b^

^a^ Chi‐square test.

^b^ Test *G*.

Analyzing the *quilombos* of the states of Pará and Tocantins together, multiple logistic regression analysis showed that the age group 12–18 (OR = 2.55; 95% CI = 1.19–5.59; *p* = 0.017), contact with an infected person (OR = 1.55; 95% CI = 1.06–2.28; *p* = 0.025), and non‐stayed in lockdown within the *quilombo* (OR = 1.82; 95% CI = 1.12–2.97; *p* = 0.016) were identified risk factors for SARS‐CoV‐2 infection when considering both states (Table S1). However, loss of taste was the only symptom statistically associated with the presence of SARS‐CoV‐2 infection (OR = 0.51; 95% CI = 0.42–0.63; *p* < 0.001).

Considering only the *quilombos* of the state of Pará, being a female (OR = 1.67; 95% CI = 1.05–2.66; *p* = 0.030), belonging to the age group of 12–18 (OR = 2.62; 95% CI = 1.20–5.7; *p* = 0.015), and rarely wearing a mask when going out (OR = 1.55; 95% CI = 1.06–2.28; *p* = 0.025) were considered risk factors for SARS‐CoV‐2 infection (Table S2). Nonetheless, staying in lockdown within the *quilombo* was significantly associated as a protective factor against infection (OR = 0.40; 95% CI = 0.21–0.79; *p* = 0.008).

In the state of Tocantins, diarrhea (OR = 2.52; 95% CI = 1.07–5.96; *p* = 0.035) and loss of taste (OR = 18.45; 95% CI = 5.02–67.85; *p* = 0.016) were significantly associated with the presence of anti‐SARS‐CoV‐2 antibodies (Table S3). Meanwhile, mask use when going out was significant and associated with protection against infection (OR = 0.36; 95% CI = 0.16–0.80; *p* = 0.012).

## 3 Discussion

The seroprevalence of anti‐SARS‐CoV‐2 IgG antibodies was estimated in *quilombos* from two states in the eastern Brazilian Amazon. This is the first seroepidemiological study conducted among these communities to describe the prevalence of anti‐SARS‐CoV‐2 IgG antibodies and analyze the characteristics of seropositive individuals from the Brazilian Amazon. Socioecological data were used to characterize social, economic, environmental, and biological aspects of the *quilombolas*, and the ecological dynamics within their territory. These indicators help to understand the health characteristics of the populations and can be used to develop more adequate public policies.

Throughout the COVID‐19 pandemic, it became evident that ethnic minorities suffered disproportionately (Bentley 2020; Tai et al. 2021). In Brazil, the seroprevalence of anti‐SARS‐CoV‐2 antibodies was strongly associated with low socioeconomic status, and a higher fatality rate was observed among the Black population (Martins‐ Filho et al. 2021; de Souza et al. 2020). In this context, *quilombolas* stand out as a group that, historically, suffered from structural racism, resulting in social exclusion, low income and education, lack of basic sanitation, difficulty in accessing health care, precarious access to water, food insecurity, high rates of infectious and chronic diseases, and other consequences of socioecological discrimination. These factors make them more susceptible to COVID‐19 (Cavalcante and Silva 2019; Carvalho et al. 2021; Oliveira and Caldeira 2016; Ferreira et al. 2022; Melo and Silva 2015; Paiva et al. 2023).

The results showed a relatively low seroprevalence of anti‐SARS‐CoV‐2 IgG antibodies in the *quilombo* communities sampled in Pará and Tocantins (40.7%), suggesting that, at the time of field research, these communities were still relatively isolated and therefore susceptible to infection. These findings differ from other studies conducted in indigenous communities in the Amazon, which reported higher seroprevalence (Lima et al. 2022; Putira Sacuena et al. 2024; Rodrigues et al. 2021). On the other hand, Martins et al. (2023) analyzed the prevalence of IgM and IgG anti‐SARS‐CoV‐2 antibodies in *quilombola* communities in the state of Sergipe, in the Northeast of Brazil, and reported seropositivity rates for IgM and IgG antibodies of 14.6% and 16.7%, respectively.

Other studies have reported lower SARS‐CoV‐2 seroprevalence in rural areas compared to urban centers (Mohanan et al. 2021; Murhekar et al. 2021). Regarding traditional communities, it has been demonstrated that the risk of infection is greater in indigenous groups when individuals resist or have difficulty adhering to recommendations for mask use and social distancing due to cultural factors (Silva et al. 2021). However, in this study, *quilombolas* generally reported following recommendations such as wearing masks and washing hands, which may have contributed to the low seroprevalence observed in some areas.

The seroprevalence of anti‐SARS‐CoV‐2 antibodies in the *quilombos* of Pará (48.3%) was higher than in Tocantins (27.3%), and both were higher than that observed in Sergipe. Although the period of COVID‐19 evolution was similar in both Amazonian states, the infection peaks were higher, and the virus spread more quickly in Pará (SESPA 2023; SES‐TO 2023), which is the most populous state in the north region (8 777 124 people) (IBGE 2022). Some studies suggest that densely populated areas can become infection centers, with potentially higher and earlier peaks of infected people (Cardoso and Gonçalves 2020; Ribeiro et al. 2020). This may have contributed to the prevalence found in Pará, even though the *quilombolas* of the state took several independent measures to try to prevent COVID‐19 from entering their territories (Carvalho et al. 2021). Conversely, given the dynamics of SARS‐CoV‐2 infection, the collection time relative to the progression of the disease in each state might also have influenced the observed prevalence.

Both in the general analysis of the two groups of *quilombos* and in the analysis only of the communities of Pará, there was a statistically significant association between the age group of 12 and 18 years and the presence of anti‐SARS‐CoV‐2 IgG antibodies (Tables S1–S3). Adolescents typically move in and out of communities for daily activities such as work, leisure, or education in more distant locations (Brasil 2018; Carril 2017) and may have been exposed to SARS‐CoV‐2 while outside the area. In Tocantins, no sociodemographic characteristics were statistically associated with infection.

In general, sociodemographic characteristics were similar when comparing seropositive *quilombos* in the two states. The analysis showed a higher prevalence of antibodies against SARS‐CoV‐2 in females as well as in individuals in the age group of 30–59 years, but without statistical significance (Table 1). In the multiple logistic regression analysis in Pará, being female was also associated with a higher risk of SARS‐CoV‐2 infection (Table S2).

In the *quilombos*, individuals of the age group 30–59 years, often the women, are the primary providers for their families. They are involved in subsistence agriculture, plant extractivism, handicraft production, manioc flower production, and selling products at local fairs and markets, or within the community, to acquire basic foodstuffs and goods. This occurs even if they are at risk of SARS‐CoV‐2 infection in crowded places (Gonçalves et al. 2021, 2023).

Although no statistically significant associations were observed in this study among income, education, self‐declared skin of color, and seropositive individuals (Table 1), the investigated communities share many similarities with others throughout Brazil. In general, the *quilombola* population mostly self‐identifies as black and brown, faces low income and education levels, poor housing, and has limited access to basic sanitation and health care, and presents high rates of death by COVID‐19 (ABRASCO 2021; Arruti et al. 2021; Ferreira et al. 2022; Santos et al. 2020; Silva et al. 2020).

Worldwide, ethnic minorities, historically socioecologically disadvantaged groups, with precarious housing, overcrowding, and difficulties in accessing health care, are disproportionately impacted by diseases (Santos et al. 2020; Tai et al. 2021). Thus, the arrival of the new coronavirus in *quilombo* communities added another layer to the suffering already experienced by this group, exemplifying a syndemic in Brazil (Gravlee 2020; Madureira and Barsaglini 2024), with consequences yet to be fully understood.

Susceptible individuals who reported direct contact with infected people had a higher seroprevalence for anti‐SARS‐CoV‐2 IgG antibodies, consistent with other studies (Li et al. 2020; To et al. 2021). Additionally, a significant association was observed between staying in lockdown within the *quilombo* and seropositivity for anti‐SARS‐CoV‐2 antibodies. This can be explained by the fact that the safe distance recommended by health authorities is difficult to apply in *quilombo* communities, where extended families often live together in small houses with limited sanitary infrastructure, and there is high interdependence among households. Therefore, housing and family clusters may contribute to the rapid spread of COVID‐19 within the community (Brasil 2022; Chan et al. 2020).

Many individuals who tested positive in both Pará and Tocantins reported using masks and washing hands (Table 2). Low educational level, lack of sanitary infrastructure, inadequate public information campaigns, and poor quality of masks represent risk factors for infection. All these factors were present in *quilombola* communities, and in association with poor access to internet, television, or radio programs, certainly limited their information about the contagion process, prevention, and prophylaxis measures. Consequently, even those who reported using masks and washing hands may not have followed correct procedures, for example, individual misunderstanding (misusing, not following guidelines) and inadequate resources such as lower quality masks and limited access to water for hand washing (Arruti et al. 2021; Del Ré et al. 2022; Scalize et al. 2021).

In general, the most frequent symptoms among the reactive individuals were headache and runny nose (Table 3), a finding similar to Martins et al. (2023). However, in the multiple logistic regression analysis, only loss of taste was statistically associated with infection in *quilombolas* in the state of Tocantins, similar to findings in other studies (Huang et al. 2020; Lee et al. 2020; Peiris et al. 2003). Furthermore, these symptoms are believed to be more frequent in patients with mild to moderate COVID‐19 infection (da Silva Torres et al. 2022; Lechien et al. 2020; Talavera et al. 2020).

There was no statistically significant association of symptoms with the presence of anti‐SARS‐CoV‐2 IgG antibodies in the *quilombos* of Pará. Knowing that during the study periods the variants in circulation in the state of Pará were Gamma (P1) and Zeta (P2), while in Tocantins, the variant in circulation was exclusively P1 (Brasil 2021), it can be assumed that the circulation of different viral variants at different times influenced the variation in COVID‐19 symptoms presented by the populations. In addition, the individual's host immune response and age may have influenced the symptomatic conditions (Dos Santos et al. 2024).

COVID‐19 is a complex disease and the most significant worldwide infectious health crisis faced by humanity in the 21st century. It has been a massive global test of human behavior in the face of biological challenges and is already the most studied disease in history. Nevertheless, much remains unknown about its manifestations and impacts across different continents, countries, and populations, particularly among rural traditional groups, which have been under‐investigated, especially due to limited funding and research capacity in developing countries.

## 4 Strengths and Limitations of the Study

The main limitations of the present study were the difficulty in obtaining samples due to social isolation decrees, making it possible to investigate only the *quilombo* communities that were included in the health actions of the Secretary of Health of Pará, and those who agreed to participate in the study in the state of Tocantins, which may have led to sample bias. Another limitation is the cross‐sectional character of the study design, which may limit the establishment of a cause‐and‐effect relationship due to the dynamics of the SARS‐CoV‐2 infection. Third, the collection time related to the progression of the disease in each state, and the cultural and environmental diversity of the *quilombos* might have influenced the observed prevalence, symptoms, and contributed to the lack of statistical associations.

However, even under very challenging geographic and pandemic conditions, we managed to obtain a representative sample of these difficult‐toto‐reach Amazonian communities which were not previously tested, of both sexes and a broad age range, and used a validated serological test which can detect past infections, including asymptomatic and recovered cases with high efficacy.

## 5 Conclusions

The COVID‐19 pandemic is proving to be one of the most devastating episodes in recent human history. The complexity of SARS‐CoV‐2 manifestations, which can vary significantly from person to person and persist for a long time, is still under investigation. However, it is already well‐established that the virus disproportionately affects the most disadvantaged populations, such as Black, *quilombola*, indigenous, and other minority groups. In Brazil and in other countries, the pandemic has exacerbated social, racial, economic, and health disparities, placing a heavy burden on health systems and challenging the capacity of health professionals (Hennis et al. 2021; Martins‐ Filho et al. 2021). Certainly, biological anthropologists, human biologists, and studies with a socioecological perspective have an important role to play both in field and laboratory research and in proposing public policies related to COVID‐19 and other health emergencies, public health programs, and vulnerable groups (Jones et al. 2021).

The low prevalence of anti‐SARS‐CoV‐2 IgG antibodies observed in the studied populations at the time of data collection highlights their epidemiological susceptibility to the COVID‐19 outbreak. Furthermore, the results demonstrate the importance of seroepidemiological studies in rural communities, as they provide information that can assist in the quick development of appropriate measures for the prevention and control of epidemic diseases in vulnerable populations. Many *quilombola* lives could have been saved if more communities had been tested during the pandemic.

To our knowledge, this was the first seroepidemiological and socioecological study of COVID‐19 in *quilombo* populations in the North region of Brazil. We hope that the information presented here can be used by the communities, the governments, and public officials in the Amazon and other regions of Brazil with a significant presence of afro‐derived communities, as well as throughout South and Central American countries with numerous traditional, black, and indigenous populations (Gates Jr. 2014; IPEA 2012). This information can help in the development of appropriate epidemic control measures tailored to the realities of these groups, especially for diseases that have transmission characteristics similar to SARS‐CoV‐2, which are likely to continue to occur in the future as global environmental changes and inequity increase in the region.

## Author Contributions

R.N.M.F., A.C.R.V., J.F.G., I.M.V.C.V., A.N.M.R.S., and J.C.M. designed and conducted the study. C.N.C.L., I.N.A., M.K.S.T., L.N.F.L., B.J.S.B., and W.F.P. assisted in subject recruitment and sample collection. Epidemiological data were entered into the database by W.R.S.B., K.A.S.P., B.C.S., V.M.E., and L.N.F.L. Experimental analyses were conducted by K.A.S.P., W.R.S.B., C.N.C.L., B.C.S., A.C.R.L., and F.T.L. Statistical analyses were performed by S.S.L. and K.A.S.P. The manuscript was written and revised by K.A.S.P., R.N.M.F., H.P.S., and A.C.R.V. All authors read and approved the final manuscript.

## Conflicts of Interest

The authors declare no conflicts of interest.

## Supporting information


**Data S1.** Supporting Information.

## Data Availability

The data that support the findings of this study are available on request from the corresponding author. The data are not publicly available due to privacy or ethical restrictions.
