# Supplementary material for: Socioecology and Prevalence of SARS‐CoV‐2 Infection in Quilombolas Living in the Brazilian Amazon
Source: Am J Hum Biol. 2025 May 3;37(5):e70055. doi: 10.1002/ajhb.70055 (PMC12048858; doi:10.1002/ajhb.70055)
Supplement: Supplementary file 1 — Data S1. Supporting Information. [file AJHB-37-e70055-s001.docx]

**SUPPLEMENTARY TABLES**

**TABLE 1:** Sociodemographic, behavioral characteristics and frequency of COVID-19 symptoms associated with the seroprevalence of SARS-CoV-2 in *quilombola* communities in the states of Pará and Tocantins.

| **Variables** | **OR**^1^ | **95% CI**^1^ | **p-value** |
| --- | --- | --- | --- |
| **Age group** |  |  |  |
| 7 to 11 | 1.06 | 0.41, 2.71 | 0.898 |
| 12 to 18 | 2.55 | 1.19, 5.59 | **0.017** |
| 19 to 29 | - | - | Ref. |
| 30 to 59 | 0.86 | 0.47, 1.61 | 0.643 |
| 60 years or older | 1.52 | 0.67, 3.46 | 0.318 |
| **Contact with infected person** |  |  |  |
| Yes | 1.55 | 1.06, 2.28 | **0.025** |
| No | - | - | Ref. |
| **Stayed in lockdown in the *quilombo*** |  |  |  |
| No | 1.82 | 1.12, 2.97 | **0.016** |
| Yes | - | - | Ref. |
| **Loss of taste** |  |  |  |
| Yes | 0.51 | 0.42, 0.63 | **< 0.001** |
| No | - | - | Ref. |
| ^1^OR = Odds Ratio, CI = Confidence Interval | | | |

**TABLE 2:** Sociodemographic, behavioral characteristics and frequency of COVID-19 symptoms associated with the seroprevalence of SARS-CoV-2 in *quilombola* communities in the state of Pará.

| **Variables** | **OR**^1^ | **95% CI**^1^ | **p-value** |
| --- | --- | --- | --- |
| **Sex** |  |  |  |
| Female | 1.67 | 1.05, 2.66 | **0.030** |
| Male | - | - | Ref. |
| **Age group** |  |  |  |
| 7 to 11 | 1,13 | 0.44, 2.91 | 0.79 |
| 12 to 18 | 2.62 | 1.2, 5.7 | **0.015** |
| 19 to 29 | - | - | Ref. |
| 30 to 59 | 0.85 | 0.45, 1.58 | 0.61 |
| 60 years or older |  |  |  |
| **Not stayed in the *quilombo* during lockdown** |  |  |  |
| Yes | - | - | Ref. |
| No | 0.40 | 0.21, 0.79 | **0.008** |
| **Use of mask when going out** |  |  |  |
| Rarely | 1.55 | 1.06, 2.28 | **0.025** |
| Always | - | - | Ref. |
| ^1^OR = Odds Ratio, CI = Confidence Interval | | | |

**TABLE 3:** Sociodemographic, behavioral characteristics and frequency of COVID-19 symptoms associated with the seroprevalence of SARS-CoV-2 in *quilombola* communities in the state of Tocantins.

| **Variables** | **OR**^1^ | **95% CI**^1^ | **p-value** |
| --- | --- | --- | --- |
| **Use of mask when going out** |  |  |  |
| Rarely | 0.36 | 0.16, 0.80 | **0.012** |
| Always | - | - | Ref. |
| **Diarrhea** |  |  |  |
| Yes | 2.52 | 1.07, 5.96 | **0.035** |
| No | - | - | Ref. |
| **Loss of taste** |  |  |  |
| Yes | 18.45 | 5.02, 67.85 | **0.016** |
| No | - | - | Ref. |
| ^1^OR = Odds Ratio, CI = Confidence Interval | | | |
